# Development and validation of a nomogram model for cancer-specific survival of patients with poorly differentiated thyroid carcinoma: A SEER database analysis

**DOI:** 10.3389/fendo.2022.882279

**Published:** 2022-09-13

**Authors:** Shuai Jin, Huiying Liu, Jingyuan Yang, Jie Zhou, Dandan Peng, Xiangmei Liu, Haiwang Zhang, Zhu Zeng, Yuan-nong Ye

**Affiliations:** ^1^ Bioinformatics and Biomedical Big Data Mining Laboratory, Key Laboratory of Infectious Immune and Antibody Engineering of Guizhou Province, Department of Medical Informatics, School of Big Health, Guizhou Medical University, Guiyang, China; ^2^ School of Public Health, Guizhou Medical University, Guiyang, China; ^3^ Department of Chronic Disease Surveillance Guizhou Center for Disease Control and Prevention, Guiyang, China; ^4^ Clinical Medical School, Guizhou Medical University, Guiyang, China; ^5^ Engineering Research Center of Cellular Immunotherapy of Guizhou Province, School of Biology and Engineering, Guizhou Medical University, Guiyang, China

**Keywords:** poorly differentiated thyroid carcinoma, nomogram, SEER database, cancer-specific survival, prognostic model

## Abstract

**Background:**

This study aimed to establish and validate an accurate prognostic model, based on demographic and clinical parameters, for predicting the cancer-specific survival (CSS) of patients with poorly differentiated thyroid carcinoma (PDTC).

**Materials and methods:**

Patients diagnosed with PDTC between 2004 to 2015 were obtained from the Surveillance, Epidemiology, and End Results (SEER) database. Randomly split the data into training and validation sets. Kaplan–Meier analysis with the log-rank test was performed to compare the survival distribution among cases. Univariate and multivariate Cox proportional hazards regression analyses were used to identify independent prognostic factors, which were subsequently utilized to construct a nomogram for predicting the 5- and 10-year cancer-specific survival of patients with PDTC. The discriminative ability and calibration of the nomogram model were assessed using the concordance index and calibration plots, respectively. In addition, we performed a decision curve analysis to assess the clinical value of the nomogram. Simultaneously, we compared the predictive performance of the nomogram model against that of the American Joint Committee on Cancer (AJCC) T-, N-, M-stage.

**Results:**

A total of 970 eligible patients were randomly assigned to either a training cohort (*n* = 679) or a validation cohort (*n* = 291). The Kaplan–Meier analysis revealed that there were no significant differences in cumulative survival based on the race, radiation, and marital status of patients. The stepwise Cox regression model showed that the model was optimal when the following five variables were included: age, tumor size, T-, N-, and M-stage. A nomogram was developed as a graphical representation of the model and exhibited good calibration and discriminative ability in the study. Compared to the T-, N-, and M-stage, the C-index of nomogram (training group: 0.807, validation group: 0.802), the areas under the receiver operating characteristic curve of the training set (5-year AUC: 0.843, 10-year AUC:0.834) and the validation set (5-year AUC:0.878, 10-year AUC:0.811), and the calibration plots of this model all exhibited better performance. At last, compared with T-, N-, and M-stage, the decision curve analysis indicated that the nomogram had excellent clinical net benefit.

**Conclusions:**

The nomogram developed by us can accurately predict the CSS of PDTC patients. It can help clinicians determine appropriate treatment strategies for poorly differentiated thyroid carcinoma patients.

## Introduction

Poorly differentiated thyroid carcinoma (PDTC) shows moderate differentiation and clinical behavior compared with well-differentiated and anaplastic thyroid carcinomas (1). In 2004, PDTC was recognized as an independent tumor entity by the World Health Organization (2). PDTC is a rare type of follicular cell-derived tumor, accounting for approximately 0.5–7% of thyroid malignancies; certain histopathological types of PDTC appear to be associated with the geographical prevalence of iodine deficiency (3, 4). PDTC is a moderately aggressive thyroid malignant tumor, with an aggressiveness between differentiated and anaplastic thyroid cancers (5). The overall survival and cancer-specific survival of PDTC is worse than that of differentiated thyroid cancer but better than that of undifferentiated thyroid cancer (6). Due to the mixed morphology and biological characteristics of PDTC, the results of iodine radiotherapy are inconsistent in different studies (7). Most patients with PDTC are treated with combination therapies, including surgery and radiation therapy. Research through univariate analysis of a small sample of data has shown that patients with PDTC distant metastasis are linked to a high mortality rate; nevertheless, there were no statistically significant differences based on the age of patients (8). Previous studies involved small sample sizes and did not analyze factors, yielding ambiguous and even contradictory findings. Therefore, a personalized prediction model for PDTC patients is needed. Nomograms are reliable and convenient prognostic tools that have been widely used to predict specific outcomes in clinical oncology.

The aim of this study was to develop a validated prognostic nomogram model for PDTC CSS to better understand the risk factors and accurately determine the prognosis. This scoring system may assist clinicians in reaching a more appropriate clinical decision. We also compared the prognostic power of this nomogram with the AJCC T-, N-, and M-stage. All data utilized in this study were drawn from the Surveillance, Epidemiology, and End Results (SEER) database (9).

## Materials and methods

### Patient selection and data processing

**Figure 1:**
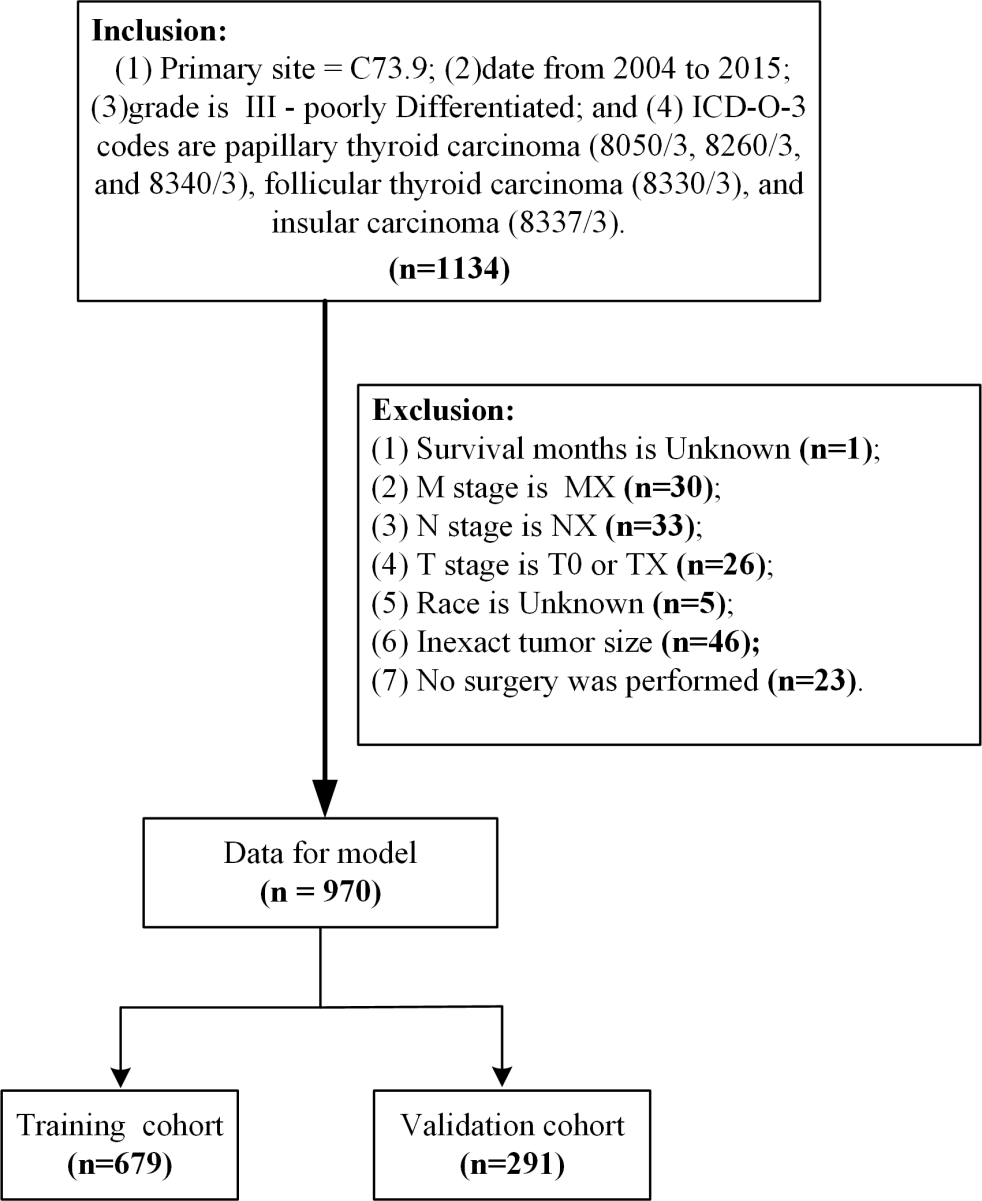
Flow chart detailing the selection of the patients in this study.

Patient data from the SEER database (Version: 8.3.9; https://seer.cancer.gov/data-software/), SEER Research Plus Data, 18 Registries, Nov 2020 Sub (2000-2018) (username for log in: 15214-Nov2020) were screened. The primary site of the thyroid gland was labeled C73.9 (10). From 2004 to 2015, a total of 1334 grade III samples were included in the study according to the International Classification of Diseases in Oncology (3rd edition) with the histological codes papillary thyroid carcinoma (8050/3, 8260/3, and 8340/3), follicular thyroid carcinoma (8330/3), and insular thyroid carcinoma (8337/3) (11, 12). The following variables were evaluated: age at diagnosis, sex, race, T stage, N stage, M stage, radiation, marital status, tumor size, survival time, and cause-specific death. The exclusion criteria in this study were: (1) unknown survival time (n=1); (2) M stage is MX (n=30); (3) N stage is NX (n=33); (4) T stage is T0 or TX (n=26); (5) race is unknown (n=5); (6) inexact tumor size (n=46); and (7) no surgery was performed (n=23). Ultimately, we identified 970 eligible patients for our study (**Figure 1**). Cancer-specific survival (CSS) was the major outcome of this study, CSS was defined as the interval from the date of PDTC diagnosis to death due to PDTC. All data from the SEER database are freely accessible for research purposes.

### Nomogram development and statistical analyses

To construct and validate the nomogram, we randomly divided the subjects into training and validation groups in a ratio of 7:3. Chi-square test for variance analysis of count data percentages. Cumulative survival time was calculated using the Kaplan–Meier method, and the differences in survival curves were analyzed using the log-rank test. The cutoff values for age and tumor size were calculated using the X-tile software (https://medicine.yale.edu/lab/rimm/research/software/) (13). Univariate and multivariate Cox proportional hazards regression analyses were performed to identify variables that significantly affected PDTC CSS. The stepwise regression based on the Akaike information criterion (AIC) minimum was used to select variables for inclusion in the nomogram. Using these identified prognostic factors, we constructed a nomogram for predicting the 5- and 10-year survival rates of patients with PDTC.

We used the concordance index (C-index) and the receiver operating characteristic curve (ROC) and determined the area under the curve (AUC) to evaluate the discriminative ability of the nomogram. Moreover, we compared the accuracy of the nomogram model with that of the T-, N-, and M-stage models. The calibration curves were used to compare the association between the actual outcomes and predicted probabilities. The clinical usefulness and benefits of the predictive model were estimated through decision curve analysis (DCA) (14). All statistical analyses were performed using R language (Version 3.6.0, https://www.r-project.org/) in the RStudio environment (Version1.2.1335, https://rstudio.com/products/rstudio/). P-values <0.05 denoted statistically significant differences.

## Results

### Patient characteristics

We included 970 PDTC patients from 2004 to 2015. The training and validation cohorts consisted of 679 and 291 cases, randomized in a 7:3 ratio. Among all PDTC patients, patients aged 11-54 accounted for 44.0%, women accounted for 61.1%, 32.5% of patients had lymph node metastasis, and 61.4% of patients had tumors between 25-84mm in size, nearly 70% of patients received radiotherapy. After being randomly divided according to the proportion, there is no difference in the percentage of each indicator between the training group and the validation group, and the two groups are comparable (**Table 1**).

**Table 1:** Demographic and clinicopathological characteristics of PDTC patients.

Characteristics	Overall	Training	Validation	χ2	p
	(N=970)	(N=679)	(N=291)		
**Age, yrs**				1.281	0.527
11-54	427 (44.0%)	297 (43.7%)	130 (44.7%)		
55-68	277 (28.6%)	189 (27.8%)	88 (30.2%)		
69-97	266 (27.4%)	193 (28.4%)	73 (25.1%)		
**Race**				0.177	0.915
Black	94 (9.7%)	67 (9.9%)	27 (9.3%)		
Other	118 (12.2%)	81 (11.9%)	37 (12.7%)		
White	758 (78.1%)	531 (78.2%)	227 (78.0%)		
**Sex**				<0.001	1.000
Female	597 (61.5%)	418 (61.6%)	179 (61.5%)		
Male	373 (38.5%)	261 (38.4%)	112 (38.5%)		
**T_stage**				1.848	0.605
T1	157 (16.2%)	104 (15.3%)	53 (18.2%)		
T2	179 (18.5%)	126 (18.6%)	53 (18.2%)		
T3	442 (45.6%)	309 (45.5%)	133 (45.7%)		
T4	192 (19.8%)	140 (20.6%)	52 (17.9%)		
**N_stage**				0.560	0.454
N0	655 (67.5%)	464 (68.3%)	191 (65.6%)		
N1	315 (32.5%)	215 (31.7%)	100 (34.4%)		
**M_stage**				0.031	0.860
M0	859 (88.6%)	600 (88.4%)	259 (89.0%)		
M1	111 (11.4%)	79 (11.6%)	32 (11.0%)		
**Tumor_size, mm**				2.755	0.252
1-24	279 (28.8%)	187 (27.5%)	92 (31.6%)		
25-84	596 (61.4%)	420 (61.9%)	176 (60.5%)		
85-348	95 (9.8%)	72 (10.6%)	23 (7.9%)		
**Radiation**				1.373	0.241
No/Unknown	296 (30.5%)	199 (29.3%)	97 (33.3%)		
Yes	674 (69.5%)	480 (70.7%)	194 (66.7%)		
**Marital**				0.625	0.429
Married	543 (56.0%)	374 (55.1%)	169 (58.1%)		
Other	427 (44.0%)	305 (44.9%)	122 (41.9%)		

Yrs, years; CI, confidence interval; CSS, cancer-speciﬁc survival; HR, hazard ratio; PDTC, poorly differentiated thyroid carcinoma.

### Associations between variables and PDTC CSS

**Figure 2:**
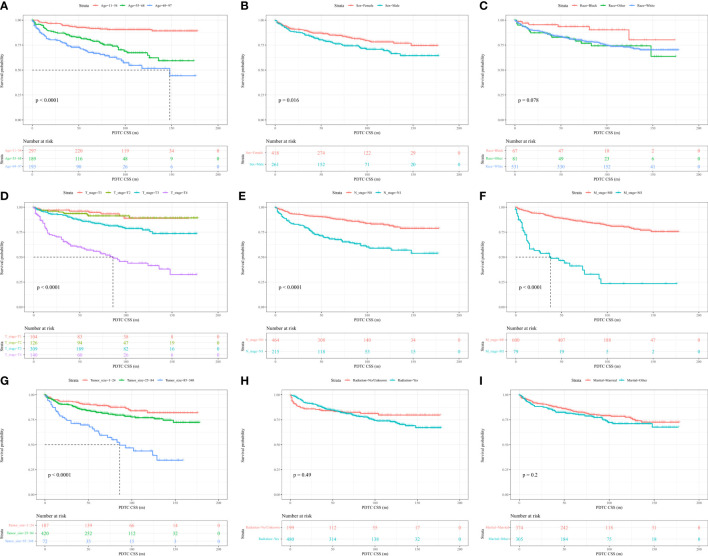
Kaplan–Meier curves and log-rank testing of cancer-specific survival (CSS) in relation to various variables in patients with poorly differentiated thyroid carcinoma (PDTC).

The log-rank test demonstrated that older age, male sex, higher level of T, N1, M1 and larger tumor sizes were linked to worse cumulative survival than other parameters (**Figures 2A, B, D–G**). Notably, there were no significant differences in the cumulative survival rate based on the race, radiation, and marital status of patients (**Figures 2C, H, I**).

### Univariate and multivariate cox regression analyses of PDTC CSS

The log-rank test and univariate Cox regression analysis gave the same conclusion, except for race. We included all variables in the multivariate Cox model, and the stepwise regression method model with the smallest AIC was the optimal model. The optimal model demonstrated that age (55–68 years: hazard ratio [HR]=2.38, P=<0.001); 69–97 years (HR=3.98, P<0.001), T2 (HR=1.08, P=0.880), T3 (HR=1.71, P=0.225), T4 (HR=3.27, P<0.009), N1 (HR=2.02, P<0.001), M1 (HR=3.89, P<0.001), bigger tumor size (85-348mm: HR=1.97, P=0.029), affected CSS in patients with PDTC. However, race, sex, radiation and marital status no longer make sense in multivariate Cox models (**Table 2**). Subsequently, a total of 5 predictors: age at diagnosis, tumor size, and T-, N-, and M-stage were utilized to construct the nomogram.

**Table 2:** Univariate and multivariate Cox regression analysis for PDTC CSS (Training Cohort).

Characteristics	Univariate analysis		Multivariate analysis	
	HR (95% CI)	p	HR (95% CI)	p
**Age, yr**				
11-54	Reference		Reference	
55-68	3.51 (2.18-5.66)	<0.001	2.38 (1.45-3.90)	<0.001
69-97	5.60 (3.54-8.87)	<0.001	3.98 (2.46-6.43)	<0.001
**Race**				
Black	Reference			
Other	2.57 (1.02-6.47)	0.045		
White	2.47 (1.09-5.61)	0.031		
**Sex**				
Female	Reference			
Male	1.50 (1.08-2.10)	0.016		
**T_stage**				
T1	Reference		Reference	
T2	1.14 (0.46-2.82)	0.784	1.08 (0.39-2.99)	0.880
T3	2.55 (1.21-5.37)	0.014	1.71 (0.72-4.05)	0.225
T4	9.06 (4.35-18.89)	<0.001	3.27 (1.34-2.92)	0.009
**N_stage**				
N0	Reference		Reference	
N1	2.85 (2.04-3.98)	<0.001	2.02 (1.40-2.92)	<0.001
**M_stage**				
M0	Reference		Reference	
M1	6.78 (4.7-9.78)	<0.001	3.89 (2.64-5.72)	<0.001
**Tumor_size, mm**				
1-24	Reference		Reference	
25-84	1.59 (1.01-2.49)	0.043	0.98 (0.58-1.68)	0.954
85-348	4.48 (2.67-7.51)	<0.001	1.97 (1.07-3.62)	0.029
**Radiation**				
No/Unknown	Reference			
Yes	1.15 (0.78-1.69)	0.491		
**Marital**				
Married	Reference			
Other	1.24 (0.89-1.73)	0.204		

Yrs, years; CI, confidence interval; CSS, cancer-specific survival; HR, hazard ratio; PDTC, poorly differentiated thyroid carcinoma.

### Nomogram construction

**Figure 3:**
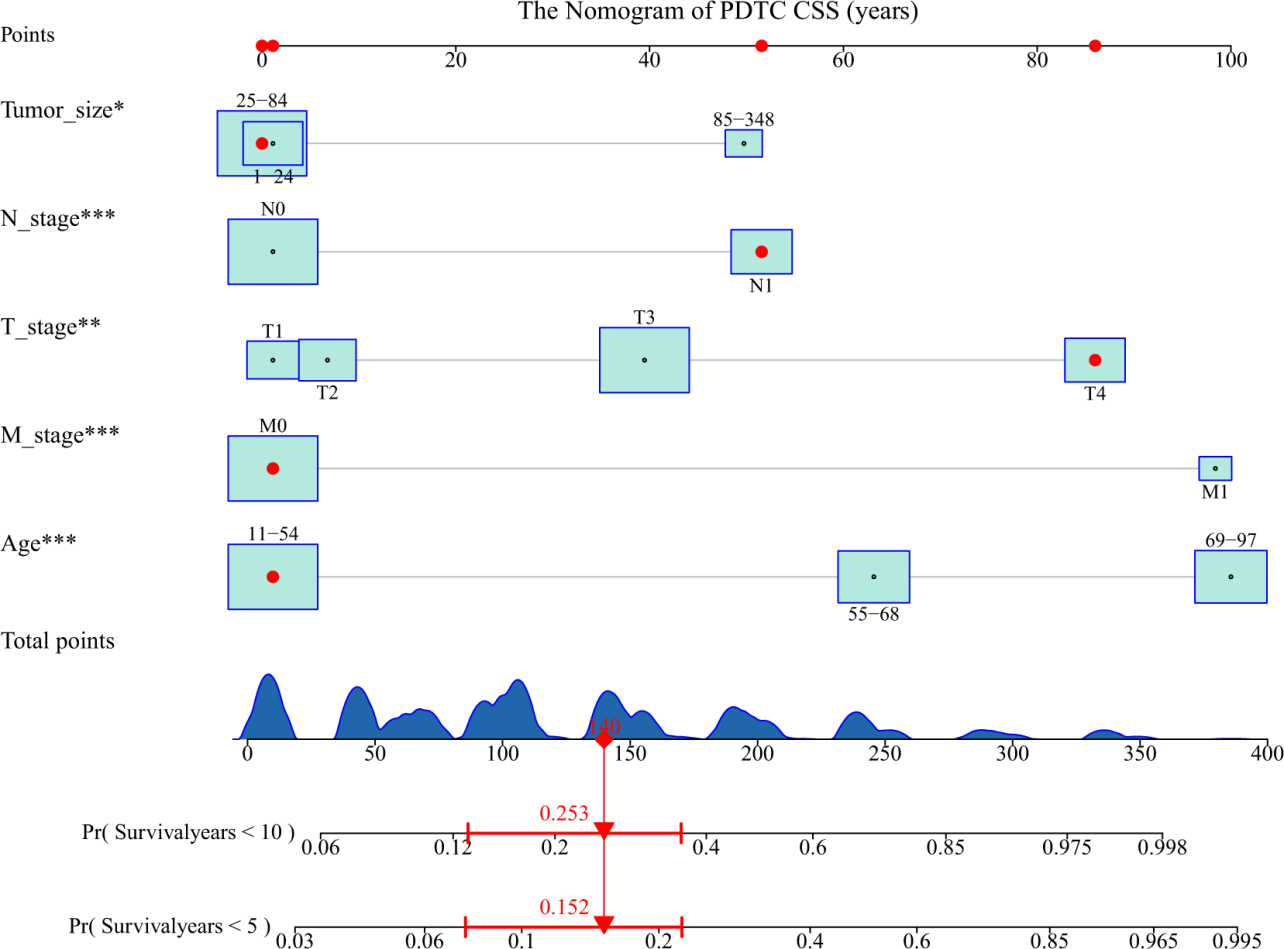
Nomogram predicting the 5- and 10-year cancer-specific survival (CSS) of patients with poorly differentiated thyroid carcinoma (PDTC) using five factors (i.e., age, T, N, M, and tumor size). The figure assumes a PDTC patient with a tumor size between 1-24 mm, lymphoid metastases, stage T4, no distant metastases, and an age between 11-54. His 5- and 10-year cancer-specific mortality rates are predicted to be 15.2% and 25.3%. * means <0.05,** means <0.01,*** means <0.001.

A nomogram based on the selected prognostic factors was developed to predict the CSS of patients with PDTC at 5 and 10 years. The nomogram demonstrated that age at diagnosis was the strongest prognostic factor, followed by M stage, T stage, N stage and tumor size. By adding the scores of all indicator levels, a total score was obtained. The prediction corresponding to this total score assisted in estimating the 5- and 10-year CSS for each patient. The nomogram results for PDTC CSS were shown in **Figure 3**.

### Validation and calibration of the nomogram

**Figure 4:**
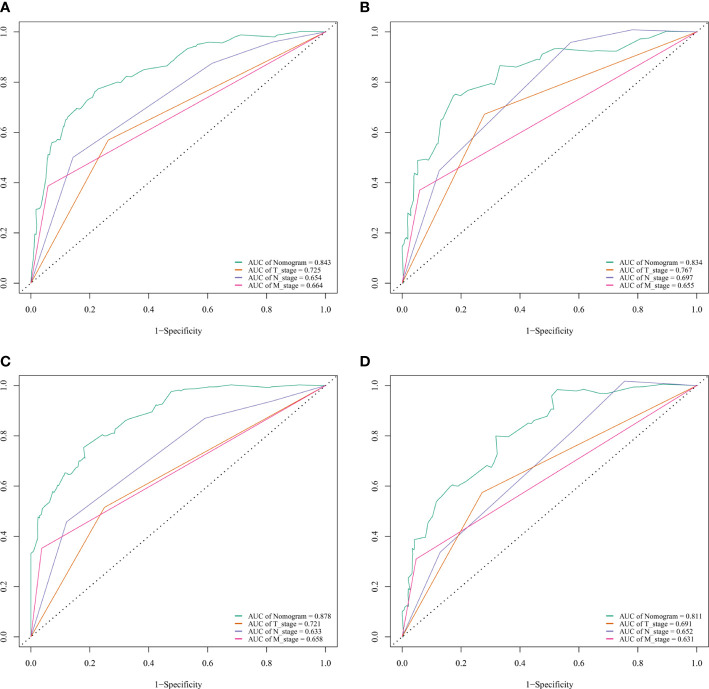
ROC curves for predictions of cancer-specific survival (CSS) in the training cohort at 5 and 10 years. ROC curves of the nomogram and AJCC T-, N-, and M-stage in training (**A**, 5-year; **C**, 10-year) and validation (**B**, 5-year; **D**, 10-year) cohorts. AJCC, American Joint Committee on Cancer; AUC, areas under the ROC curve; ROC, receiver operating characteristic curve; TNM, tumor–node–metastasis.

**Figure 5:**
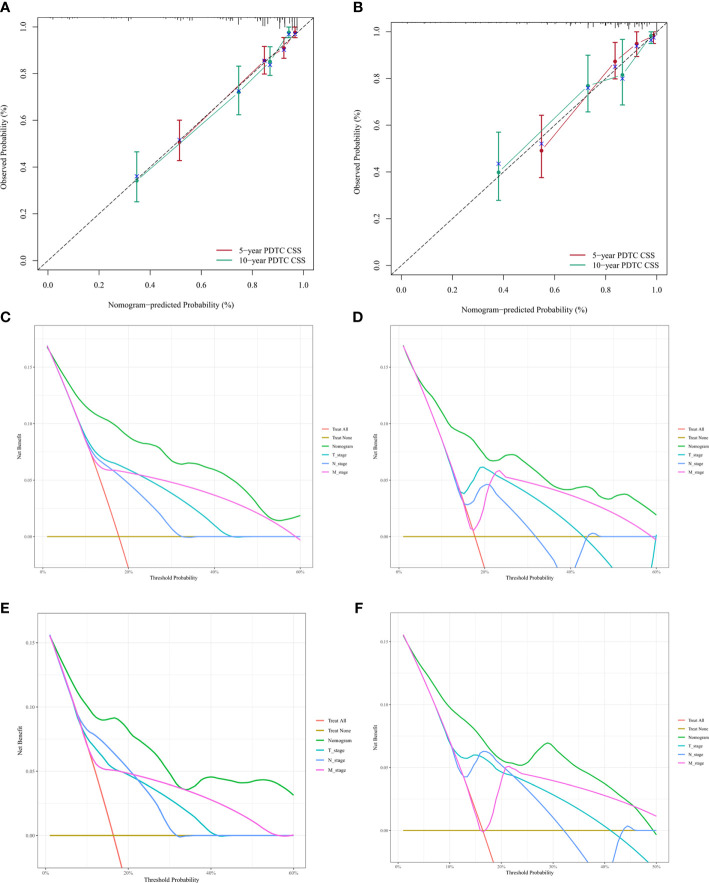
Calibration plots and decision curve analysis for predictions of cancer-specific survival (CSS) in the training and validation cohort at 5 and 10 years.Calibration plots of the nomogram in training **(A)** and validitaion **(B)** cohorts. Decision curve analysis for the Nomogram and AJCC T-, N-, and M-stage in prediction of prognosis in training (**C**, 5-year; **D**, 10-year) and validation (**E**, 5-year; **F**, 10-year) cohorts.

The C-index of nomogram (training group: 0.807, validation group: 0.802). Furthermore, our model showed better discriminative ability compared to the traditional T-, N-, and M-stage in both training (5-year AUC: 0.843 vs. 0.725 vs. 0.654 vs. 0.664 (**Figure 4A**), 10-year AUC: 0.878 vs. 0.721 vs. 0.633 vs. 0.658 (**Figure 4C**) and validation (5-year AUC: 0.834 vs. 0.767 vs. 0.0.697 vs. 0.655 (**Figure 4B**), 10-year AUC: 0.811 vs. 0.691 vs. 0.652 vs. 0.631 (**Figure 4D**) cohorts for 5- and 10-year PDTC CSS. The calibration plots indicate the level of agreement between the predicted value of the nomogram and the actual value for the CSS of patients with PDTC. In this study, the calibration plots of nomogram showed good agreement between the actual observations and the predicted outcomes both in the training (**Figure 5A**) and validation cohorts (**Figure 5B**) for 5- and 10-year PDTC CSS.

Finally, DCA was conducted to compare the clinical utilities and benefits of the nomogram and T-, N-, and M-stage with regard to the CSS of patients with PDTC. These analyses at 5 and 10 years showed greater net benefits across a range of mortality risks for the nomogram compared with that of the T-, N-, and M-stage both in training (**Figures 5C, D**) and validation (**Figures 5E, F** **)** cohorts. These results confirmed that the nomogram was more practical than the AJCC T-, N-, and M-stage.

## Discussion

Thyroid cancer is one of the common malignant tumors in the endocrine system (15). However, few studies have investigated PDTC; public understanding of its clinical features continues to be unclear, and treatment and prognostic factors remain controversial. Therefore, it is necessary to establish a risk prediction model for the CSS of patients with PDTC, which will assist in the development of treatment strategies for patients with PDTC. The AJCC T-, N-, and M-stage are useful in determining the prognosis of patients with thyroid cancer, it may ignore risk factors. In this study, we developed a comprehensive nomogram model based on the combination of various risk factors to better predict the CSS of patients with PDTC. The nomogram model developed in this study, which included age, T-, N-, and M-stage, and tumor size, was able to more accurately predict the CSS of PDTC compared with the AJCC T-, N-, and M-stage.

Patients with PDTC have a lower survival rate versus those diagnosed with papillary and medullary thyroid carcinoma (16, 17). Therefore, we analyzed CSS-related features in patients with PDTC. In the present study, the ratio of females to males was 1.57:1 (18). CSS was significantly worse in males than in females; however, this difference did not remain significant in the multivariate analysis. Old age is currently recognized as a predictor of poor prognosis for numerous diseases (19). Through multivariate analysis, Asioli et al. (20) confirmed the negative age-related predictions for patients with PDTC. The eighth edition of the AJCC Cancer Staging Manual defines 55 years of age as an important point for staging (21), and the results of the study demonstrate the importance of this age point. In addition, this study found that if PDTC patients were older than 68 years, they had lower CSS than patients in other age groups. Kong et al. studied the relationship between age and cancer-specific prognosis of anaplastic thyroid cancer, and they confirmed that cancer-specific prognosis was poorer in patients over 70 years of age (22). These results provide a useful addition to the tumor staging manual.

PDTC is highly aggressive (5, 23) and typically diagnosed at an advanced stage with extra-glandular proliferation and extensive local infiltration. The regional lymph node and distant metastasis rates are 30–59% and 13–33%, respectively (24). This study demonstrated that N1 is a risk factor in both univariate and multivariate analyses; these findings are consistent with those reported by Christelle et al. (25). M0 is a protective factor for CSS in PDTC patients; this is in accordance with data reported by Ibrahimpasic et al. (18). The T-stage is often used to evaluate the prognosis of tumors. In this study, the T stage was shown to be a prognostic factor, and the survival of patients with T3 and T4 disease was worse than that of patients with T1 and T2 disease. At present, the treatment decision for PDTC is mainly based on previous experience in the treatment of differentiated thyroid cancer (18). Hiltzik et al. found that iodine intake was not associated with PDTC overall survival (26). The study demonstrated no benefit from radiation therapy on PDTC cancer-specific survival. This may be due to the fact that PDTC is often seen in advanced age and late stage, both of which are more aggressive biological manifestations and therefore may lead to loss of RAI avidity. The role of postoperative external irradiation therapy (EBRT) in PDTC is equally controversial (18). In this study, the sample of patients who performed BEAM radiation was small and was not analyzed as a distinct level.

Compared with the T-, N-, and M-stage, the nomogram established in this study has better discriminative ability and accuracy for predicting the 5- and 10-year CSS of patients with PDTC. In addition, using verification and calibration, it was confirmed that the nomogram more accurately predicted survival versus the AJCC T-, N- and M-stage (27). The present model may directly help physicians to assess the risk of cancer-related death and, consequently, devise appropriate treatment strategies for patients with PDTC.

This study also has several limitations. Firstly, this was a retrospective study without dynamic control. Secondly, in the sample selection process, we excluded patients with missing data on the collected variables, which may have led to selection bias. Thirdly, the samples were obtained from the SEER database and external validation was not performed. In view of the deficiencies of retrospective research, it is necessary to carry out large-scale prospective randomized controlled trials.

## Conclusion

To better determine the CSS of patients with PDTC, we have developed and validated a nomogram for predicting the 5- and 10-year CSS based on a large cohort with real-world samples. The proposed nomogram considered five independent risk factors: age, T, N, M, and tumor size. Comparison with the AJCC T-, N-, and M-stage, this nomogram confirmed excellent discriminative ability and potential clinical application.

## Data availability statement

The datasets presented in this study can be found in online repositories. The names of the repository/repositories and accession number(s) can be found in the article/supplementary material.

## Ethics statement

Ethical review and approval was not required for the human participants because the SEER database is free for researchers to download and has removed patient information.

## Author contributions

ZZ, Y-NY, and SJ were responsible for the conception and design of the study. SJ, JYY, and HWZ collected data and did the statistical analysis. HYL, DDP, XML, and JZ wrote and revised the manuscript. All authors have read and approved the final version of the manuscript.

## Funding

This work was supported by the National Natural Science Foundation of China (NO.32160151, 61803112), and the NSFC Incubation Program by Guizhou Medical University (20NSP033), and the Science and Technology Foundation of Guizhou Province (NO.2019-2811).

## Conflict of interest

The authors declare that the research was conducted in the absence of any commercial or financial relationships that could be construed as a potential conflict of interest.

## Publisher’s note

All claims expressed in this article are solely those of the authors and do not necessarily represent those of their affiliated organizations, or those of the publisher, the editors and the reviewers. Any product that may be evaluated in this article, or claim that may be made by its manufacturer, is not guaranteed or endorsed by the publisher.
